# Comparative Review on Cancer Pathology from Aberrant Histone Chaperone Activity

**DOI:** 10.3390/ijms25126403

**Published:** 2024-06-10

**Authors:** Jiho Lee, Xiucong Bao

**Affiliations:** School of Biomedical Sciences, Faculty of Medicine, The University of Hong Kong, Hong Kong SAR, China; jhlee01@connect.hku.hk

**Keywords:** histone chaperone, cancer therapy, targeted therapies

## Abstract

Histone chaperones are integral to chromatin dynamics, facilitating the assembly and disassembly of nucleosomes, thereby playing a crucial role in regulating gene expression and maintaining genomic stability. Moreover, they prevent aberrant histone interactions prior to chromatin assembly. Disruption in histone chaperone function may result in genomic instability, which is implicated in pathogenesis. This review aims to elucidate the role of histone chaperones in cancer pathologies and explore their potential as therapeutic targets. Histone chaperones have been found to be dysregulated in various cancers, with alterations in expression levels, mutations, or aberrant interactions leading to tumorigenesis and cancer progression. In addition, this review intends to highlight the molecular mechanisms of interactions between histone chaperones and oncogenic factors, underscoring their roles in cancer cell survival and proliferation. The dysregulation of histone chaperones is significantly correlated with cancer development, establishing them as active contributors to cancer pathology and viable targets for therapeutic intervention. This review advocates for continued research into histone chaperone-targeted therapies, which hold potential for precision medicine in oncology. Future advancements in understanding chaperone functions and interactions are anticipated to lead to novel cancer treatments, enhancing patient care and outcomes.

## 1. Introduction

DNA stores genetic information. Chromatin, a complex of DNA and proteins, plays a crucial role in organizing and regulating genetic information. Nucleosomes, the basic units of chromatin, each contain 147 base pairs of DNA wrapping around an octamer of histone proteins—two histone H2A/H2B dimers and one histone H3/H4 tetramer [1,2]. These histone proteins provide structural support to compact chromatin structures through DNA condensation [3]. Nucleosomes interacting with histone proteins continue assembly and disassembly by the deposition or eviction of histone proteins, which is modulated by several mechanisms such as ATP-dependent chromatin remodeling, histone post-translational modifications, and the incorporation of histone variants [4,5]. The dynamic nature of chromatin structures allows the fine-tuning of gene expression in response to various cellular signals and environmental impacts.

Histone chaperones were first identified in the 1980s and are reported to play a significant role in chromatin dynamics by facilitating nucleosome assembly and disassembly [6]. Unlike other histone-binding proteins, histone chaperones do not exhibit enzymatic activity and act in a histone-specific manner [7]. To date, dozens of histone chaperones have been identified, each with distinct biological functions, including prevention of non-specific interaction with DNA, promotion of their proper deposition/eviction, and transcriptional regulation [8]. Histone chaperones’ activity and their correlation with cellular processes highlight their critical role in genome integrity maintenance.

Research on histone chaperones has significantly progressed over the past few decades, through advancements made with various methodologies. Initial studies employed biochemical fractionation and in vitro reconstruction assays to characterize the specific binding between chaperone proteins and histone proteins [9,10,11]. Further advancements in molecular biology and proteomics techniques—such as co-immunoprecipitation, mass spectrometry, and cross-linking—have facilitated the identification of proteins possessing histone chaperone activities [10,11,12]. Additionally, approaches made through structural biology—including cross-linking mass spectrometry, X-ray crystallography, and cryo-electron microscopy—have provided molecular insights into the chaperoning mechanism mediating nucleosome assembly and disassembly [13].

**Figure 1:**
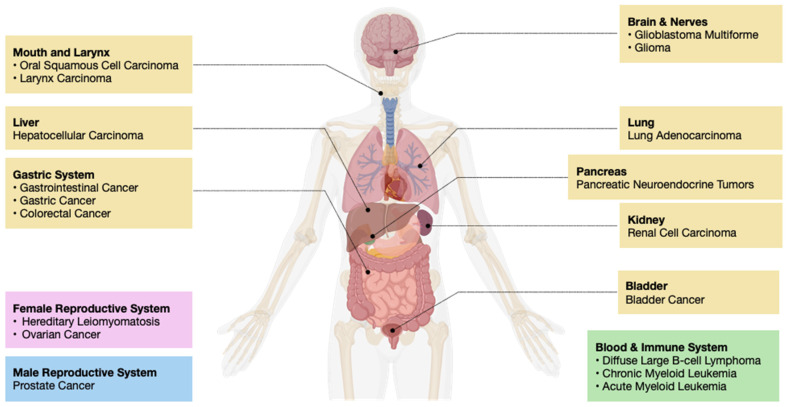
The impact of histone chaperone malfunctioning in cancer: Abnormal expression or activity of histone chaperones can lead to several types of carcinogenesis and tumorigenesis. These can occur in anatomically distinct regions of the body, such as the brain, mouth, larynx, liver, pancreas, lung, kidney, bladder, gastric system, and blood, as well as sex-specific organs such as the prostate and the uterus.

The dysregulation of histone chaperones has shown a high correlation with various cancer pathologies, highlighting the potential of histone chaperone proteins as a therapeutic target (Figure 1, Table 1). Alterations in histone chaperone expression levels, mutations, or aberrant interactions with other cellular factors can lead to disruptions in chromatin structure and gene expression, ultimately driving tumorigenesis and cancer progression [14]. Furthermore, certain histone chaperones have been shown to interact with oncogenic transcription factors or chromatin modifiers, thereby promoting cancer cell survival and proliferation [15]. Given their significant roles in modulating chromatin dynamics and impacting gene regulation, histone chaperones have emerged as promising therapeutic targets in cancer therapy. Small molecules and peptidomimetic inhibitors have been developed to target specific histone chaperones or their interactions with histones, aiming to disrupt their functions and restore normal chromatin architecture [16,17,18]. These targeted therapies hold promise for the development of personalized cancer treatments and the potential to overcome resistance mechanisms associated with current chemotherapeutic strategies [19]. In this review, we will discuss the molecular mechanisms of histone chaperones and their implications in carcinogenesis.

## 2. Results

### 2.1. FACT Complex

The FACT complex is a heterodimeric protein complex, containing SSRP1 (Structure-Specific Recognition Protein 1) and SPT16 (Suppressor of Ty 16), that has been identified as a histone chaperone protein [20]. FACT has been reported to play a crucial role in chromatin regulation, impacting essential DNA-dependent processes like DNA damage repair and DNA replication [21,22,23]. FACT interacts with RNA polymerase II elongation, facilitating its breakage of the nucleosomal barrier [24]. Moreover, FACT recognizes and binds to specific histone post-translational modifications (methylation, acetylation, phosphorylation, glutarylation, sumoylation, ubiquitination, etc.) and histone variants (H2AX, Macro H2A, CENP-A, H2B1A, H2AZ, etc.), which enables it to regulate the chromatin landscape [21]. The FACT complex is reported to be over-expressed in several hypoxic cancers and can provide more insight into therapeutic approaches.

#### 2.1.1. Human Hepatocellular Carcinoma (HCC) and FACT

HCC is the sixth most common and second most fatal cancer worldwide [25]. Hypoxic microenvironments containing reactive oxygen have been shown to induce HCC tumor development [26]. Despite its prevalence and active treatment approaches, many HCC patients suffer from chemoresistance and drug resistance, which highlights an urgent need to develop molecular targets to treat HCC [27].

**Figure 2:**
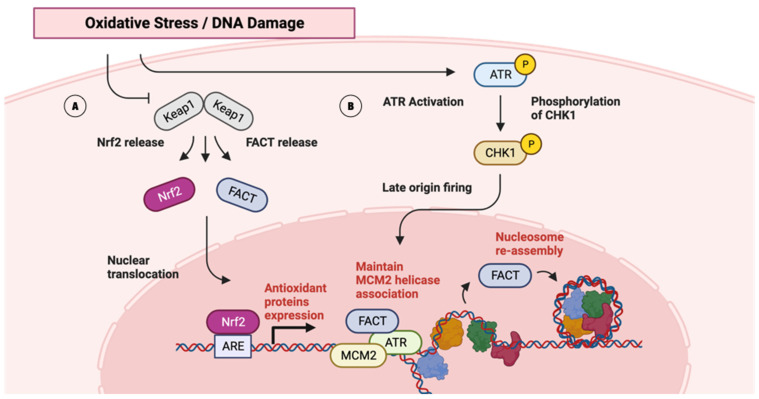
FACT complex-associated pathways. (**A**) Potential implication of FACT complex in NRF2/KEAP1 pathway. FACT complex works in tandem with NRF2, establishing a positive feedback loop to over-express antioxidant proteins [31]. (**B**) A potential implication of the FACT complex in the ATR/CHK1 pathway is to prevent replication stress in response to DNA damage [22,30].

Elevated levels of the FACT complex facilitate the growth and metastasis of HCC, as evidenced by CRISPR-mediated gene manipulation, which underscores its role in HCC proliferation [21]. Its involvement in the oxidative stress response is particularly notable; the FACT complex works in tandem with NRF2—a master regulator of the antioxidant response—to evade KEAP1-mediated degradation (Figure 2A) [21]. This synergy between FACT and NRF2 establishes a positive feedback loop, crucial for the transcriptional activation of antioxidant defense genes, thereby propelling the cancer’s survival and aggression [21].

#### 2.1.2. Breast Cancer (BrCa) and FACT

BrCa is the most common cancer among women worldwide, accounting for 25% of all cancer cases and causing significant morbidity and mortality [28]. Despite advances in early detection and treatment, BrCa remains the leading cause of cancer-related death among women, highlighting the need for a better understanding of the underlying molecular mechanisms and improved identification of novel therapeutic targets [29].

Laura Prendergast et. al. revealed that not only are proliferating tumor cells with high replication stress sensitive to FACT expression, but the absence of FACT during replication stress also leads MCM2-7 helicase to dissociate from chromatin [22]. FACT’s essential function is to maintain the association of the MCM2-7 helicase with chromatin, thereby safeguarding the replication fork and enabling the ATR/CHK1 pathway—which stabilizes replication forks and prevents DNA damage—in response to replication stress (Figure 2B) [22,30]. This checkpoint activation is vital for stabilizing stalled replication forks and preventing excessive origin firing, the absence of which, particularly in FACT-depleted scenarios, culminates in DNA damage accumulation and subsequent cell death [22].

#### 2.1.3. FACT-Targeted Therapy: Curaxin

Defining the FACT complex as a novel target for cancer treatment enabled researchers to investigate Curaxin as a therapeutic treatment for HCC patients [32]. Curaxin, a small molecular inhibitor of FACT that induces chromatin trapping of FACT and significantly inhibits normal human FACT activities, causes intracellular acidification and apoptosis of cancer cells [31,33]. Curaxin treatment has been shown to block the gene expressions needed for oxidative stress and hypoxia responses in HCC cells, presumably in correlation with the FACT complex’s interaction with NRF2 [34]. Moreover, breast cancer cells with different FACT complex expression levels were sensitive to Curaxin treatment in vitro [34].

### 2.2. ASF1

ASF1A and ASF1B are mammalian-specific paralogous genes, a gene type that has been widely reported as a histone chaperone protein [35]. Both ASF1A and ASF1B are involved in various chromatin-associated processes, such as DNA replication, damage repair, and gene transcription, through interaction with histone H3-H4 tetramer [36]. Although ASF1A and ASF1B share a high degree of sequence similarity and can bind histone H3-H4 tetramer with similar affinity, they have been shown to have distinct cellular functions and expression patterns [37]. Moreover, ASF1A and ASF1B interact with other histone chaperone proteins, fostering the chromatin regulation processes [38]. Over-expression or depletion of the ASF1A and ASF1B proteins is reported to be associated with carcinogenesis and tumorigenesis through interaction with other histone chaperone proteins [39,40].

#### 2.2.1. Lung Adenocarcinoma (LUAD) and ASF1

LUAD is the most prominent type of total non-small cell lung cancer, comprising at least 40% of the total lung cancer count. Despite the new therapies developed to treat this prominent pathogenetic symptom, the 5-year survival rate is under 15%, highlighting the significance of deciphering its molecular mechanisms and identifying novel therapeutic targets [41].

**Figure 3:**
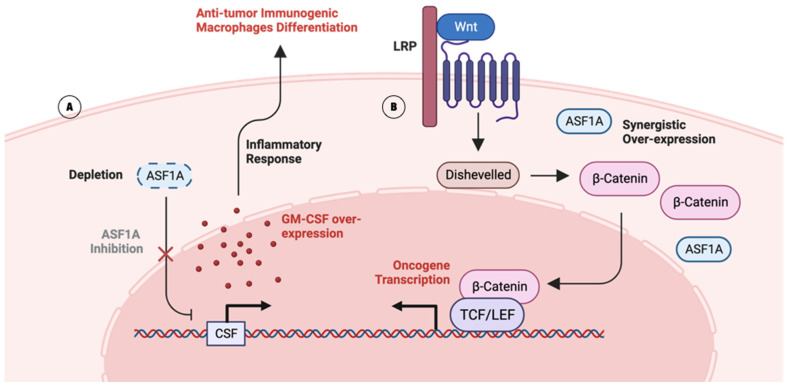
ASF1 complex-associated pathways. (**A**) Potential implication of ASF1A in GM-CSF-dependent inflammatory pathway. ASF1A inhibits the over-expression of GM-CSF. ASF1A depletion (dashed box) blocks (red cross) the inhibition pathway, which leads to the over-expression of GM-CSF, establishing an anti-tumor immunogenic response [32]. (**B**) The potential implication of ASF1A in Wnt signaling pathway is to cause the over-expression of β-catenin, which stimulates oncogene transcription [40].

Fei Li et. al. identified ASF1A as a promising molecular target for *Kras*-mutant LUAD—a major oncogenic driver accounting for 32% of all LUAD patients [42]. Before the identification, *Kras*-mutant LUAD had unsatisfactory response rates to immunotherapy [43]. Fei Li et. al. used in vivo CRISPR screening to reveal that the intrinsic ASF1A deficiency in LUAD cells upregulates GM-CSF expression, which induces the differentiation of immunogenic macrophages in the microenvironment (Figure 3A) [42,44]. ASF1A is known to control the acetylation level of histone, while histone acetylation is enriched at the promoter region of GM-CSF in immunogenic T-cells [35,45]. In addition to ASF1A inhibition, the combinatory application of immune checkpoint blockade anti-PD1 assists in stronger T-cell activation [42].

Although the role of ASF1B in the tumorigenesis of LUAD has not been shown clearly, Wencheng Zhang et. al. found that downregulation of ASF1B has a mechanistic implication in LUAD progression through the indirect regulation of proteins, including POLE3 and CKS1B, which disrupts the integrity of chromatin dynamics [46,47,48].

#### 2.2.2. Gastrointestinal Cancer (GIC) and ASF1

GIC encompasses a group of malignancies that affect the digestive system, including the esophagus, stomach, liver, pancreas, gallbladder, and intestines [49]. Globally, GIC accounts for significant morbidity and mortality, with stomach and colorectal cancers being among the top five causes of cancer-related deaths [50]. Given the substantial global burden of GIC, there is a need to decipher its underlying molecular mechanisms and seek potential therapeutic targets. 

ASF1A is over-expressed in GIC cells, synergistically with β-catenin—a key component of the Wnt signaling pathway (Figure 3B) [40]. β-catenin plays a crucial role in transmitting signals from cell surface receptors to the nucleus, where it regulates the transcription of target genes involved in cell proliferation and survival, such as c-MYC and cyclin D1 [51]. Stimulated oncogene transcription can stimulate the proliferation, stemness, migration, and invasion of GIC cells [40]. 

Moreover, through the incorporation of various GIC cell lines with multiple analyses, ASF1B has been hypothesized to control the cell cycle and promote cell proliferation, as it has shown involvement in S phase and M phase activities, such as DNA replication or nuclear and other organelle division [52]. Knockdown of ASF1B prevented GIC cells from progressing from the G0/G1 phase to the S phase, which possibly results from impaired chromatin assembly due to the depletion of key histone chaperones [52].

#### 2.2.3. ASF1-Targeted Therapy Approach: Chimeric Inhibitor

Defining ASF1 as a novel therapeutic target led researchers to seek molecular inhibitors to induce the depletion of cancerous tissue, including LUAD and GIC tissues. For example, Johanne Mbianda et. al. reported their discovery of a foldamer inhibitor of ASF1 to inhibit its protein–protein interaction [38]. Johanne used oligoureas—which have shown resistance to plasma degradation—to mimic the α-helix that specifically binds to the ASF1 histone 3 binding domain [38]. Moreover, there are multiple approaches to screening ASF1-inhibiting small molecules, including binding assays [18]. Through binding assays, Ja-Hwan Seol et. al. identified pyrimidine-2,4,6-trione (PYT) derivatives that successfully inhibited the interaction between ASF1 and histone H3 [18]. Greg F Miknis et. al. identified a series of N-acryl hydrazones acting as small molecule inhibitors inhibiting the interaction between the ASF1-H3/H4 complex [53].

### 2.3. APLF

APLF is a scaffold protein that has been identified as a histone chaperone protein [54]. APLF has been reported to play a crucial role in maintaining chromatin integrity as a non-homologous end-joining DNA repair factor that promotes impaired chromatin repair of DNA-damaged sites [55]. APLF uses its acidic DNA-binding domain (APLFAD) to bind with histone complexes [56]. Specifically, APLFAD obtains two aromatic side chains that preferentially attach to α1–α2 patches on histone H2A–H2B dimer, which supports its chaperone activity torward H2A–H2B [56]. Recently, APLFAD has been reported to assemble nucleosomes much faster than canonical nucleosome assembly processes [57].

#### 2.3.1. Glioblastoma Multiforme (GBM) and APLF

GBM is the most common type of adult brain cancer, with rapid growth and aggressiveness [58]. GBM patients have very poor prognoses, with an average of 14 and 16 months of life expectancy after the first diagnosis [58]. Although there have been significant efforts to seek a cure for GBM with advancements in treatment methods for brain cancer, the survival rate of GBM patients has not yet improved, which is due to the low sensitivity of GBM toward temozolomide (TMZ) chemotherapy and radiotherapy [59,60]. There is an urgent need to find a molecular target for the effective treatment of GBM.

**Figure 4:**
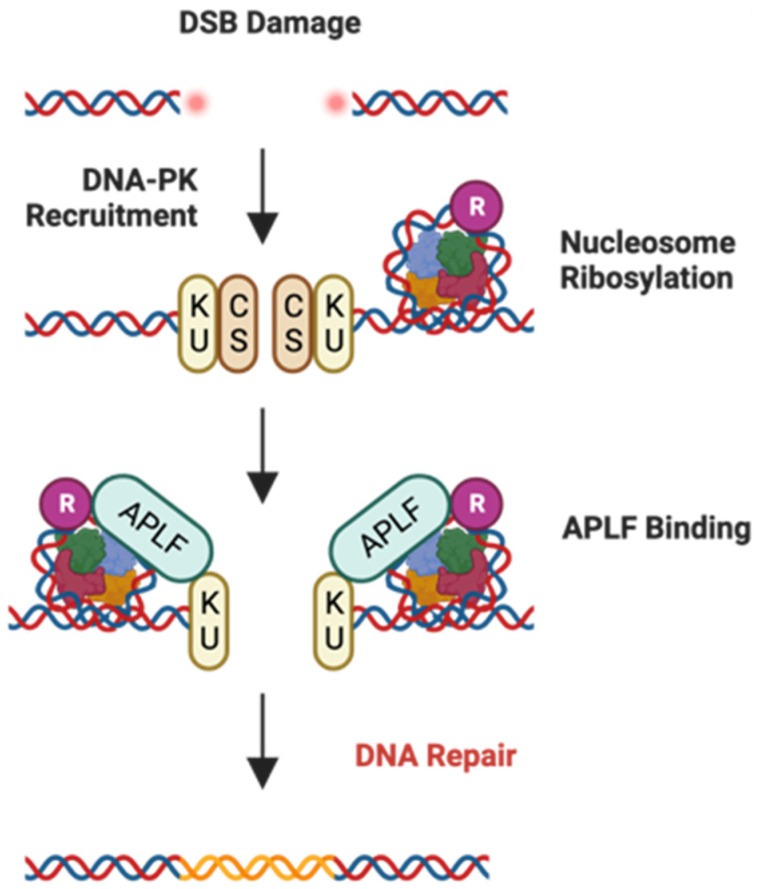
APLF-associated pathway. Potential implication of APLF in NHEJ pathway. APLF interacts with KU and ribosylated nucleosometo establish DNA repair after double-strand break damage [62]. R: ribosylation. Blue, green, orange and red colors: histones.

APLF expression is positively correlated with GBM patients’ resistance toward TMZ treatment [61]. APLF, as a histone chaperone, maintains genome integrity by promoting the recruitment of other proteins required for non-homologous end joining (NHEJ) [55]. In fact, in TMZ treatment-resistant or radiotherapy-resistant GBM cells, a significant increase in the speed of the DNA repair process by the NHEJ pathway has been observed, while APLF contributes to NHEJ efficiency by interacting with KU components (Figure 4) [61,62]. Furthermore, APLF deficiencies in GBM cells and mouse models have demonstrated that it may lead TMZ resistance to be overcome and induce apoptosis [61,62].

#### 2.3.2. Bladder Cancer (BLCA) and APLF

BLCA is the fourth most common cancer in the male population, with a recurrence rate of around 40% [63]. Female patients diagnosed with bladder cancer tend to have more advanced forms of BLCA, due to their lack of perception toward symptoms of BLCA [64]. Although current treatment methodologies for BLCA are promising, patients with invasive BLCA—such as muscle-invasive bladder cancer (MIBC)—have a high chance of recurrence, which highlights the need to decipher the molecular mechanisms of BLCA for effective treatment [65,66]. One characteristic of MIBC is genomic instability caused by dysfunctional DNA double-strand break (DSB) repair, including the NHEJ pathway [67]. In comparison to other, non-invasive BLCA species, Christin Richter et. al. reported that patients suffering from MIBC show high transcription factor E2F1 levels [67].

High E2F1 levels transactivate factors associated not only with NHEJ but also with MIR888, which indirectly represses APLF, upon hypo-methylation in invasive BLCA species [67]. APLF repression by ‘out of context’ activity of MIR888 in BLCA causes perturbations in the chromatin assembly and repair process, causing an increased risk of cell invasiveness [67].

#### 2.3.3. APLF and Treatment Approaches

Defining APLF as a novel therapeutic target is necessary for treating cancers caused by abnormal over-expression or suppression of the DNA repair processes of the NHEJ pathway. Therefore, Christin Richter et. al. sought methods to restore the NHEJ pathway [67]. They reported that depletion of MIR888 can restore the supply of APLF for proper NHEJ function, which is favorable for BLCA patient survival [67].

### 2.4. NPM1

NPM1 is a nucleolar phosphoprotein, participating in the transcriptional regulation mechanism, that has been identified as a histone chaperone protein [68,69]. NPM1 has been reported to be involved in chromatin-related processes, such as nucleosome assembly, chromatin remodeling, and ribosome biogenesis [70]. NPM1 interacts with other proteins, including the Ran-Crm1 complex, synergistically regulates nucleosome assembly, and affects other histone chaperones’ functional activity [71]. Moreover, NPM1’s two weak nuclear export signal (NES) motifs and its amphoteric characteristics provide structural support to its histone chaperone activity [71]. NPM1’s role in various biological mechanisms makes it relevant to the study of various diseases, such as cancer.

#### 2.4.1. Oral Squamous Cell Carcinoma (OSCC) and NPM1

OSCC is a prevalent type of malignant cancer occurring in the mouth, characterized by high morbidity and mortality rates [72]. There have been significant advances in treatment strategies that have caused the 5-year survival rate to improve to 60% in the last few decades [73]. However, late diagnosis of OSCC causes poor outcomes, which emphasizes the need for a better understanding of the molecular mechanisms underlying OSCC development and progression [73,74].

In OSCC, the total pool of canonical NPM1 and acetylated NPM1 (AcNPM1) is increased, highlighting that NPM1 and AcNPM1’s interactions with other proteins—such as histone acetyltransferase, chromatin remodelers, RNA Polymerase II, and transcription factors—and their histone chaperone activity contribute to OSCC development and proliferation [69]. Meanwhile, NPM1’s depletion and the subsequent decrease in AcNPM1 occupancy abrogates tumor growth and reduces the gene transcription required for OSCC proliferation and invasive metastasis [69].

#### 2.4.2. Acute Myeloid Leukemia (AML) and NPM1

AML is one of the most aggressive and prevalent cancers of the myeloid line of blood cells, accounting for 80% of all adult leukemia cases and with a 28% rate of 5-year survival [75,76]. Recent advancements in managing AML have improved the survival rate greatly, reaching up to 40% in patients with an age below 60 [77]. However, patients with an age over 60 still have a low survival rate. In fact, AML patients who undergo initial treatment with high-intensity chemotherapy regimens still need to take post-remission therapy, which highlights the need to decipher its molecular mechanisms for the effective treatment of AML patients [78].

**Figure 5:**
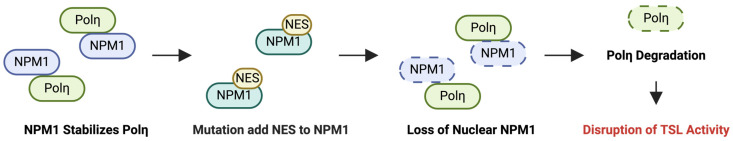
NPM1-associated pathway. A potential implication of NPM1 in the DDT pathway is to stabilize Polη,necessary for TSL activity [83]. The addition of NES to NPM1 leads to the loss of nuclear NPM1, thereby resulting in Polη degradation and TSL activity disruption.

The C-terminus of NPM1 (NPMc+) is observed in more than 50% of adult AML cases [79]. This mutation causes the removal of the nucleolar localization signal (NLS) but adds a nuclear export sequence (NES) to NPM1, which causes the loss of nuclear NPM1 [80,81,82]. This phenomenon has been widely reported to cause degradation of Polη—a Y-family polymerase that is necessary for translesion synthesis (TSL) activity for DNA damage tolerance (DDT) in cells (Figure 5) [83]. Polη degradation disrupts cytarabine, a chemotherapeutic drug for AML therapy, preventing Polη’s participation in the DNA damage repair process [84].

#### 2.4.3. NPM1-Targeted Therapy: NSC348884 and RNAi

Defining NPM1 as a diagnostic factor has allowed physicians to detect various pathologies early. Moreover, NPM1 is defined as novel therapeutic target, and there have been multiple approaches to inhibit NPM1 for effective cancer treatment. Targeting NPM1 oligomerization directly may increase the effectiveness of tretinoin and cytarabine therapy in NPM1c+-expressing AML cells. Ramesh Balusu et. al. used two methods to target NPM1 oligomerization: shRNA knockdown and NSC348884 NPM1 inhibitor [85]. Both the shRNA and the NSC348884 method abolished the lethal AML phenotype, caused apoptosis, and sensitized AML cells to tretinoin and cytarabine [85]. Moreover, another study reports that NSC348884 can induce apoptosis in Ewing sarcoma cancer [86].

### 2.5. CAF-1

**Figure 6:**
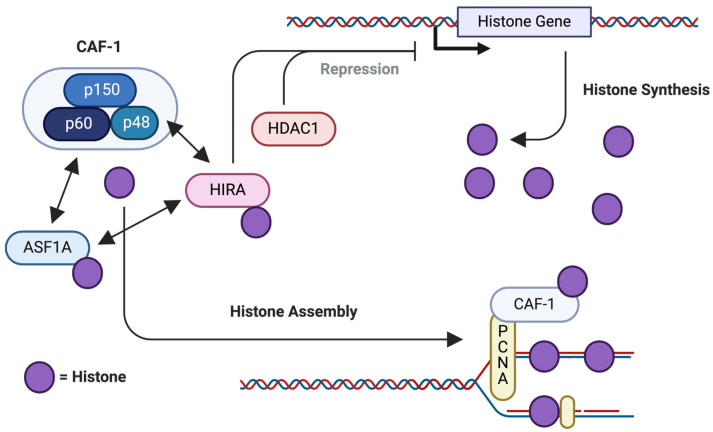
The CAF-1-associated pathway. CAF-1 plays a significant role in the deposition of newly translated histone on DNA [152]. It is crucial in maintaining cell viability and, thus, is a tumor proliferation marker [155].

CAF-1 is a histone chaperone that primarily interacts with histones H3–H4, playing an essential role in DNA replication-coupled nucleosome assembly [150]. CAF-1 is a heterotrimeric protein complex composed of p150, p60, and p48 subunits, which together regulate the deposition of newly synthesized histones onto newly replicated DNA (Figure 6) [151,152]. Within the subunits of CAF-1, the p48 subunit has been reported to play a critical role in packaging DNA into nucleosomes [153]. On the other hand, p150 (CHAF1A) and p60 (CHAF1B) have been extensively reported in regard to their histone chaperoning activities [154]. The dysregulation of canonical CHAF1A, CHAF1B, and p48 subunits’ expressions or functions has been implicated in several diseases, including cancer and viral infections that require their DNA replication-coupled nucleosome assembly [82].

#### 2.5.1. Gastric Cancer (GC) and CHAF1A

GC remains a significant global health problem, ranking as the third leading cause of cancer-related deaths worldwide [87]. Despite advances in diagnostic and therapeutic strategies, the mortality rate remains high, primarily due to late-stage diagnosis and a high incidence of recurrence after treatment [87]. In this context, the histone chaperone CHAF1A—one of the subunits of histone chaperone CAF-1—has emerged as a crucial player in GC pathology. CHAF1A has been found to promote GC tumor growth, potentially through its influence on the chromatin landscape and consequent gene expression profile [88].

High expression levels of CHAF1A might be linked with poor disease-free survival and overall survival rates in non-cardia GC patients undergoing fluoropyrimidines-based adjuvant chemotherapy post-radical gastrectomy [89]. Deqiang Wang et. al. suggested that CHAF1A may affect chemotherapy outcomes by interacting with the folate metabolism pathway and manipulating the expression of thymidylate synthetase (TS), a key enzyme in DNA synthesis and repair [89]. Given these findings, CHAF1A represents a promising avenue for the development of novel therapeutic strategies and predictive biomarkers in GC, potentially contributing to improved patient prognosis and survival.

#### 2.5.2. Diffuse Large B-Cell Lymphoma (DLBCL) and CHAF1A

DLBCL represents the most common subtype of non-Hodgkin’s lymphoma, contributing to a significant portion of lymphoma-related mortality globally [90]. Despite advances in therapeutic options, the heterogeneous nature of DLBCL and the lack of specific molecular targets pose a formidable challenge to its management [91].

An aberrant epigenetic axis involving the histone chaperone CHAF1A in the context of DLBCL has been identified. Wei Yan et. al. reported that high expression levels of CHAF1A, as revealed by bioinformatic analysis and validated in patient samples, were associated with poorer prognoses and shorter survival periods in DLBCL patients [92]. This elevated CHAF1A expression contributed to DLBCL aggressiveness, including enhanced cell proliferation, migration, and in vivo growth. In summary, the histone chaperoning activity of CHAF1A and its influence on the epigenetic landscape and autophagy processes play a significant role in DLBCL pathogenesis and represent a promising direction for improving DLBCL management.

#### 2.5.3. Larynx Carcinoma, Skin Squamous Cell Carcinomas (SCCs), and CHAF1B

Larynx carcinoma and skin SCCs represent significant health burdens globally, with high morbidity and mortality rates [93,94]. Their development and progression are driven by complex molecular changes, among which epigenetic alterations play a crucial role. Therefore, it is crucial to discover novel biomarkers for larynx carcinoma and skin SCCs.

Massimo Mascolo et. al. employed tissue microarray (TMA) technology for high-throughput evaluation of CHAF1B expression across various cancer types, including, for the first time, larynx carcinoma and skin SCCs [95]. As a result, over-expression of CHAF1B was observed in both malignancies, with the highest levels of expression associated with more aggressive and metastasizing tumors [95]. These findings underscore the potential of CAF-1 p60 as a prognostic marker in larynx carcinoma and skin SCCs, offering new insights into their pathogenesis and indicating promising directions for targeted therapeutic strategies.

#### 2.5.4. SCC and CHAF1B

SCCs represent a diverse group of malignancies arising from squamous epithelial cells, often associated with significant mortality and morbidity [96]. Among these, there is a particular interest in the SCC of the tongue due to its aggressive behavior and poor prognosis [97]. The complex molecular landscape of SCCs involves key epigenetic alterations, among which the role of CHAF1B has been highlighted.

Stefania Staibano et. al. examined the immunohistochemical expressions of CHAF1B and CHAF1A in a series of tongue SCCs [98]. Over-expression of CHAF1B was observed in all the tumors, whereas CHAF1A was downregulated in a number of cases [98]. Notably, the over-expression of CHAF1B and the downregulation of CHAF1A were associated with poor prognosis, beyond classical prognostic parameters [98]. These findings suggest that a simultaneous deregulation of cell proliferation and DNA repair, mediated by CAF-1, is present in aggressive tongue SCCs. The evaluation of CHAF1B expression might serve as a valuable tool for assessing the biological behavior of these tumors, potentially guiding improved follow-up protocols and the development of alternative therapeutic strategies.

#### 2.5.5. CC and p48

Cervical cancer (CC) is the fourth most common cancer among the female population worldwide [99]. CC is induced by persistent infection with human papillomavirus (HPV), which is one of the most common sexually transmitted infections [100]. There are several different factors that contribute to the prognosis of CC, such as age, race, disease stage, etc. Although there are multiple treatment options available, patients with advanced-stage CC and elderly patients tend to have a low 5-year survival rate of below 50% [99]. This highlights the critical need to understand CC’s molecular pathogenesis and discover innovative therapeutic strategies and diagnostic factors.

Li Kong et al. identified p48 as a crucial mediator in the interaction between human papillomavirus (HPV) and cervical cancer (CC) [101]. Knockdown experiments demonstrated that suppressing p48 significantly increased cell proliferation and enhanced the transformation activity of HPV16, whereas over-expression of p48 inhibited CC tumor formation [101]. These findings indicate that p48 plays an essential role in regulating the progression of CC, particularly in modulating the oncogenic activity of HPV16. Additionally, Estelle Nicolas et. al. has reported an association between p48 and the histone deacetylase complex (HDAC), noting that inhibition of HDAC can stimulate HPV16 gene expression [102,103]. These observations suggest that p48 may be involved in the molecular pathogenesis of CC and could potentially enhance patient prognosis and survival as a novel therapeutic target.

#### 2.5.6. THCC and p48

Thyroid carcinomas (THCC) represent malignant cells around thyroid tissue [104]. The incidence of THCC is continuously increasing, accounting for 1% to 4% of all malignancies diagnosed each year [104]. The prognosis of THCC highly depends on the type of cell. Most THCCs are papillary, which is highly treatable [104]. However, aggressive THCCs, such as anaplastic (ATC), are highly malignant and have an extremely low survival rate of 6 months after diagnosis, regardless of the treatment [105]. This underscores the critical need to understand ATC’s molecular pathogenesis and develop novel therapeutic strategies.

Studies have indicated that inhibiting NF-κB in ATC enhances the chemotherapeutic response [106]. Francesco Pacifico et. al. demonstrated a robust correlation between p48 and NF-κB in ATC via differential proteomic analysis, revealing that p48 expression decreases in the absence of NF-κB [156]. Furthermore, silencing p48 expression through siRNA has been shown to reduce tumorigenicity and increase apoptotic activity [156]. Previous research has identified NF-κB as a regulator of apoptosis and contributor to chemoresistance, acting in conjunction with Akt1 through the downregulation of histone H4 expression [157]. Concurrently, p48 has been shown to interact with histone H4 independently of the other subunits of CAF-1 [158]. These findings suggest a possible link between the histone chaperoning activity of p48 and cancer pathogenesis, providing new perspectives on targeting p48 for the treatment of ATC.

#### 2.5.7. CAF1-Targeted Therapy Approach: CRISPR/Cas9

Defining CAF-1 as a novel therapeutic target is necessary for treating cancers caused by abnormal over-expression and for treating cancer patients with chemo-resistance from aberrant CAF-1 expression. Inhibition of CAF-1 is reported to induce intrinsic immunity [159]. The depletion of CAF-1 induces high enrichment of histone H3.3, which increases chromatin accessibility and endogenous retrovirus element (ERV) expression. STING and dsRNA viral sensing pathways then sense high expression of ERV activating anti-tumor immune surveillance.

### 2.6. HIRA

The HIRA complex is an evolutionarily conserved histone chaperone, which has been widely reported as a critical chromatin regulator in senescent cells [107]. HIRA interacts with histone variants (such as H3.3) and supervises their deposition to promote active replication-independent transcription [108]. Moreover, other histone chaperones assist HIRA in performing multiple activities: 1. DNA damage repair, 2. H3.3 enrichment regulation, 3. myogenic progression, etc [109]. Depletion of HIRA is reported to be associated with carcinogenesis and tumorigenesis through interaction with histone variants and other histone chaperones [110].

#### 2.6.1. Hereditary Leiomyomatosis and Renal Cell Carcinoma (HLRCC) and HIRA

HLRCC is a rare and aggressive from of renal cancer caused by germline mutations in the fumarate hydratase (FH) gene, which encodes a mitochondrial enzyme involved in the tricarboxylic acid (TCA) cycle [111]. Due to its distinctive molecular pathogenesis, unveiling its biomarkers is crucial for HLRCC patients [112].

**Figure 7:**
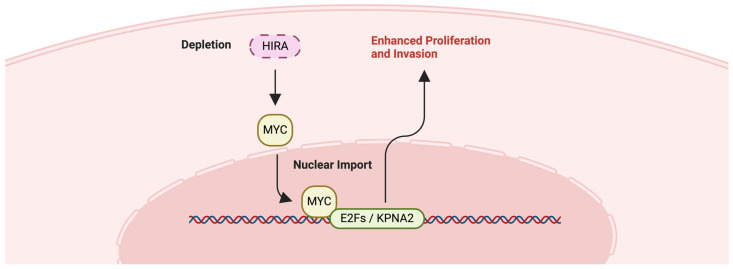
HIRA-associated pathway. Potential implication of HIRA in MYC pathway. Depletion (dash box) of HIRA induces nuclear import of MYC, activating target oncogenes and enhancing proliferation and invasion of cancer cells [114].

Fred H. Menko et. al. revealed a novel, non-canonical role of HIRA in HLRCC, through genome-wide CRISPR-Cas9 screening [113]. The loss of HIRA enhances the proliferation and invasion of FH-deficient cells, both in vitro and in vivo [113]. Moreover, HIRA deficiency activates the proto-oncogene MYC and its target genes, leading to increased nucleotide metabolism specifically in FH-deficient cells (Figure 7) [114]. This suggests that HIRA can regulate oncogenic processes in a manner that extends beyond its traditional role in chromatin dynamics. Thus, HIRA’s involvement in HLRCC progression underscores the complex interplay between genetic and epigenetic regulation in cancer and highlights the potential of targeting the HIRA-MYC pathway for the treatment of HLRCC.

#### 2.6.2. Chronic Myeloid Leukemia (CML) and HIRA

CML is a rare form of leukemia involving the over-production of myeloblasts [115]. Moreover, more than 95% of CML patients have the Philadelphia chromosome, a distinctive cytogenetic abnormality [116]. Although the currently available frontline therapies for CML provide descent prognoses, there are still cases of frontline therapy failure [117]. Therefore, identifying new pathways is crucial for providing additional therapy options for CML patients.

Aditi Majumder et. al. revealed that the expression of HIRA is upregulated in CML cells compared to normal cells [118]. Knockdown experiments on the K562 cell line using shRNAs demonstrated that the depletion of HIRA led to limited proliferation but increased differentiation, which indicates that HIRA directly interacts with specific genes involved in cell cycle regulation and differentiation—suggesting its role in controlling gene expression patterns in CML [118]. Mechanistically, HIRA-sh cells show ectopic enrichment of histone H3.3 in the MKL1 and GATA2 promoters, but its loss in the GYPA promoter, contributes to the megakaryocyte differentiation [118]. Thus, HIRA or its associated pathways could potentially be explored as a therapeutic target for CML treatment.

#### 2.6.3. HIRA-Targeted Therapy Approach: shRNA

Defining HIRA or its related pathways as a novel therapeutic target is necessary for treating patients for whom the frontline therapies have failed. Ana P. Gomes et. al. highlighted HIRA as a therapeutic target [160]. The downregulation of HIRA by shRNA induced the suppression of cancer aggressiveness-inducing transcription factors, which hindered metastasis in the breast cancer cell line LM2 and in immunocompromised mice [160].

### 2.7. NAP1

NAP1 and its family proteins—including NAP1-like proteins—have been extensively studied for their role in chromatin assembly and remodeling [119,120]. Research has shown that NAP1 interacts with histones and functions as a histone chaperone, assisting in the assembly of nucleosomes. It assembles nucleosomes by inhibiting non-nucleosomal DNA–histone interaction [120]. NAP1L1 specifically has been implicated in the promotion of cell proliferation and the progression of the cell cycle, particularly from the G1 phase to the S phase [121]. Moreover, NAP1 and NAP1-related proteins have the ability to bind to the H2A-H2B dimer and the H3-H4 tetramer, highlighting their significance in chromatin landscape regulation [153].

#### 2.7.1. Glioma and NAP1L1

Glioma is a type of brain tumor that arises from glial cells, which are supporting cells of the central nervous system [122]. Chemotherapy may be administered orally or intravenously and can be used in combination with radiation therapy to enhance effectiveness [123]. However, the prognosis for glioma patients remains challenging. Ongoing research aims to identify new therapeutic targets and develop more effective treatment strategies for glioma patients.

Data from The Cancer Genome Atlas (TCGA) and immunohistochemical analyses revealed that NAP1L1 is upregulated in glioma tissues, with its expression being positively correlated with aggressive clinical features such as higher WHO grades, KPS, Ki-67 index, and recurrence rates [121]. NAP1L1’s interaction with hepatoma-derived growth factor (HDGF) appears to be a linchpin in this association. Together, they activate c-Jun, a potent oncogenic transcription factor that upregulates the cell cycle promoters CCND1/CDK4/CDK6, thus, driving cell proliferation and contributing to cisplatin therapy resistance [121]. This interaction not only elucidates a mechanism by which these proteins promote tumorigenesis but also positions NAP1L1 as a potential biomarker and therapeutic target in glioma.

#### 2.7.2. Ovarian Cancer (OVCA) and NAP1L1

OVCA is the most lethal gynecological malignancy and often goes undetected until it reaches advanced stages [124,125]. Symptoms in the early stages are often vague and nonspecific, which contributes to the challenges of early diagnosis [126]. Treatment options for OVCA include surgery, chemotherapy, and in some cases, targeted therapy [125]. However, despite those advancements, the high rate of recurrence and the development of resistance to chemotherapy pose significant challenges [127].

**Figure 8:**
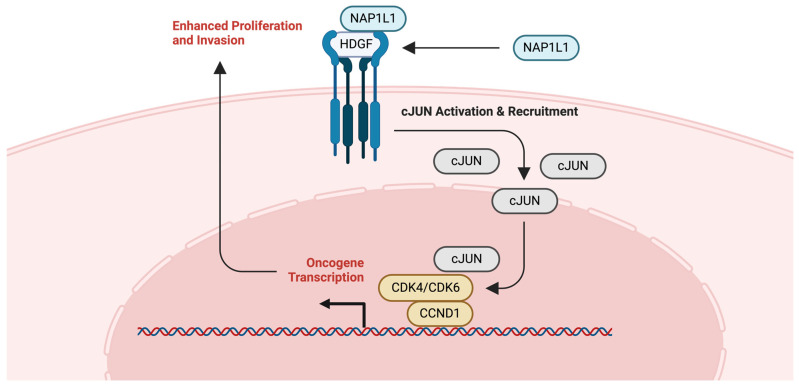
NAP1L1-associated pathway. Potential implication of NAP1L1 in JNK signaling pathway. Interactions between NAP1L1 and HDGF activate cJUN and thereby activate oncogene transcription [128].

Functional assays demonstrate that a reduction in NAP1L1 expression in OC cell lines results in suppressed proliferation, cell cycle arrest, and increased apoptosis, reinforcing its role in oncogenesis [128]. NAP1L1’s interaction with HDGF and their subsequent co-localization in the cytoplasm leads to the activation of the C-JUN transcription factor and the upregulation of CCND1, promoting cell cycle progression (Figure 8) [128]. The histone chaperoning activity of NAP1L1 could lead to the over-expression of oncogenes such as C-JUN and CCND1 or the repression of tumor suppressor genes, further promoting ovarian cancer progression. Therefore, these findings suggest NAP1L1’s multifaceted role in OVCA pathophysiology and the potential for targeted therapeutic interventions.

#### 2.7.3. NAP1L1-Targeted Therapy: Gene Therapy Approach

Defining NAP1L1 as a novel therapeutic target is necessary for treating cancers caused by abnormal over-expression of oncogenes. YaHui Liu et. al. reported that targeting the NAP1L1 histone chaperone induces the reduction of oncogene transcription [161]. The reduced expression of NAP1L1 induces suppression of HDGF recruitment and its expression, which induces reduced expression of C-JUN and CCDN1 [161].

### 2.8. SPT6

SPT6 is an essential histone chaperone that plays a pivotal role in the precise regulation of transcriptional elongation [129]. SPT6 tightly associates with nucleosomes, aiding in the reassembly of nucleosomes during gene transcription [129]. Using its tandem Src2 homology domain, SPT6 shows interaction with RNA polymerase II, which is expected to control mRNA turnover during transcription facilitated by SPT6 [129]. In addition, SPT6 recognizes and binds to the H3-H4 tetramer, and collaboratively works with the H2A–H2B dimer-binding FACT complex [130].

#### 2.8.1. Colorectal Cancer (CRC) and SPT6

CRC is the third most commonly diagnosed cancer globally and the third leading cause of cancer-related death [131]. It originates within the colon or rectum, often from benign polyps that can, over time, undergo malignant transformation [132]. The prognosis for CRC is heavily dependent on the stage at diagnosis, with early-stage detection offering a ~90% chance of 5-year survival [116,133]. This underscores the critical need for an enhanced understanding of CRC’s molecular pathogenesis and the development of innovative therapeutic strategies.

**Figure 9:**
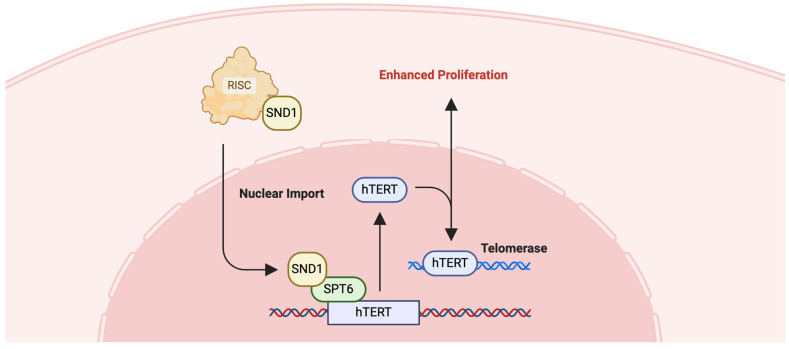
SPT6-associated pathway. Potential implication of SPT6 in hTERT signaling pathway. SND1, part of the RISC complex, interacts with SPT6, which establishes transcription of hTERT [134].

Chaoliang Diao et. al. discovered that SPT6 synergistically interacts with staphylococcal nuclease and Tudor domain containing 1 (SND1) and co-control hTERT expression, which is pivotal for the limitless replicative potential of cancer cells, a hallmark of CRC (Figure 9) [134]. Knockdown experiments have shown that depleting SPT6 expression not only diminishes hTERT levels, but also curtails CRC cell proliferation, invasion, and stem-like characteristics, while simultaneously increasing apoptotic activity and sensitivity to chemotherapy [134]. This shows that SPT6 can be a promising avenue for the development of novel therapeutic strategies for CRC, potentially contributing to improved patient prognosis and survival.

#### 2.8.2. SPT6-Targeted Therapy: Chaetocin

Defining SPT6 as a novel target for cancer treatment enabled researchers to identify chaetocin. Chaetocin, a non-specific inhibitor of histone lysine methyl-transferases, mimics SPT6 loss at the transcriptome level when used in treatment [162]. In vivo tests on mice reported that chaetocin treatment not only reduced tumor sizes but also significantly extended survival [162]. Moreover, it was identified that chaetocin treatment can lead to reductions in endogenous SPT6 protein levels, causing DSB and apoptosis of tumor cells [162].

### 2.9. DAXX

DAXX functions as a multifaceted histone chaperone, implicated in the deposition of histone H3.3 at telomeric and pericentric chromatin, influencing gene expression and chromatin structure [135]. Beyond its traditional role in apoptosis, DAXX plays a crucial role in genome stability by maintaining the structural landscape of global heterochromatin [136]. Moreover, recent discoveries suggest that DAXX recruits histone methyltransferases, promoting H3K9me3 catalysis prior to their deposition [137].

#### 2.9.1. Pancreatic Neuroendocrine Tumors (PanNETs) and DAXX

PanNETs are neoplasms arising from the hormone-producing cells of the pancreas, accounting for less than 5% of all pancreatic cancers [138]. Although PanNETs are generally more slow-growing than pancreatic adenocarcinomas, they can be aggressive and metastatic [139]. The clinical presentation of PanNETs can vary, with some tumors functioning to produce endocrine hormones, while others are nonfunctioning [139]. Despite surgical resection and available therapies, advanced PanNETs have a poor prognosis, underscoring the necessity of understanding the molecular pathways that govern PanNET pathogenesis.

**Figure 10:**
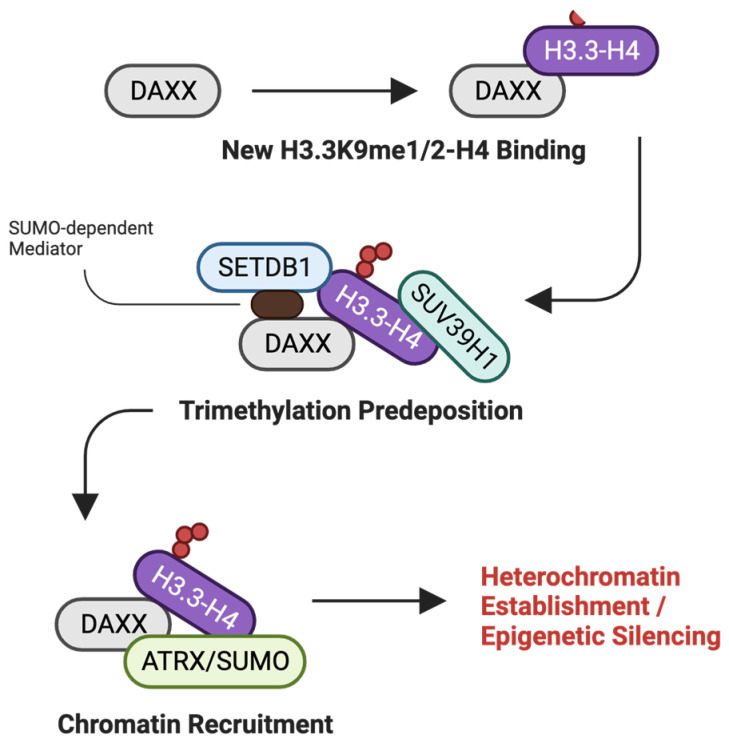
DAXX-associated pathway. Potential implication of DAXX in epigenetic silencing pathway to recruit chromatin through H3.3K9 trimethylation deposition [137,140].

Hitoki Ueda et al. reported that the loss of DAXX protein expression is crucial for PanNET pathogenesis, highlighting the importance of its regulation of chromatin architecture and gene expression [140]. Mechanistically, frequent somatic mutations cause DAXX to lose its canonical transcriptional repressor function, leading to the dysregulation of key genes implicated in PanNET progression [140]. Moreover, functional studies involving DAXX knockdown and knockout in PanNET cell lines have demonstrated that DAXX exerts its tumor-suppressive effects by forming a complex with histone H3.3 and promoting trimethylation of lysine 9 on histone H3 (H3K9me3), a modification associated with gene silencing (Figure 10) [140]. Therefore, the histone chaperoning activity of DAXX and its influence on the epigenetic landscape suggest a promising therapeutic strategy for PanNET patients.

#### 2.9.2. Prostate Cancer (PCa) and DAXX

PCa is the second most frequently diagnosed cancer in the male population and the fifth leading cause of mortality globally [141]. Characterized by the abnormal growth of cells in the prostate gland, PCa development is often fueled by androgen hormones [142]. The complexity of PCa’s progression and the heterogeneity of its response to treatment necessitate ongoing research to elucidate the molecular underpinnings of the disease and to develop innovative treatment strategies that can improve long-term patient survival [143].

Genome-wide ChIP-Seq analysis of PC3 prostate cancer cells has revealed an extensive network of over 59,000 DAXX binding sites, predominantly localized at enhancers and promoters of active regulatory elements, underscoring the broad impact of DAXX on the prostate cancer epigenome [144]. Notably, DAXX recruitment is associated with the localization of DNA methyltransferase 1 (DNMT1), a key partner in the epigenetic silencing machinery; however, DNMT1’s binding is more selectively confined to a subset of these sites [144]. The binding pattern suggests that DAXX may play a role in guiding DNMT1 to specific genomic loci, reinforcing the repression of target genes, including those involved in autophagy [144].

#### 2.9.3. DAXX-Targeted Therapy Approach

Defining DAXX as a novel therapeutic target is necessary for treating cancers caused by abnormal regulation of key regulatory genes. The stability of the global heterochromatin landscape that APLF maintains is well understood, although defining it as a therapeutic target is still difficult due to our lack of mechanistic understanding. Therefore, there is an urgent need to fill this research gap.

### 2.10. C1QBP

C1QBP is a doughnut-shaped mitochondrial protein that has been reported as a key player in immune response and oxidative phosphorylation [145,146]. Recently, Jianwei Lin et. al. proposed that C1QBP acts as a histone chaperone, recognizing the tails of histones H3 and H4 [147]. Although various diseases, such as breast cancer and renal cancer, have shown irregular C1QBP expression, the exact mechanisms and pathways through which C1QBP functions as a histone chaperone remain understudied [148,149]. Therefore, there is an urgent need to fill this research gap to gain a clearer understanding of its mechanisms.

## 3. Discussion

The field of histone chaperone research holds great promise for the future of cancer therapy. As our understanding of the molecular mechanisms dictating chaperone function deepens, so too will our ability to craft precise interventions. The exploration of small molecule inhibitors, such as Curaxin, provides a framework for the development of targeted therapies aimed at specific chaperones or their interactions. The potential to integrate these novel agents with existing treatments promises a more effective and personalized approach to cancer care. Additionally, ongoing advancements in molecular biology and structural analysis techniques will undoubtedly yield new insights into histone chaperone biology, catalyzing the discovery of next-generation cancer therapeutics. The horizon for histone chaperone-targeted therapies is expansive, offering a beacon of hope for improved patient outcomes in the battle against cancer.

## 4. Conclusions

In conclusion, the extensive research on histone chaperones underscores their pivotal role in preserving genomic and epigenomic stability by modulating the dynamic eviction and deposition of histones. The dysregulation of these chaperones has been inextricably linked to the pathogenesis of various cancers, as evidenced by their aberrant expression and function in tumor development and progression. The insights gleaned from studies on histone chaperones illuminate the intricate balance between chromatin dynamics and cellular homeostasis. The discovery of histone chaperone-targeted therapies exemplifies the potential of targeting histone chaperones to disrupt cancer cell survival mechanisms. Moreover, these findings suggest that histone chaperones are not merely bystanders, but active participants in cancer pathology, presenting a fertile ground for therapeutic innovation.

## Figures and Tables

**Table 1:** Summary of all histone chaperones and their associated pathways, cancer implications, and targeted therapy approaches.

Histone Chaperones	Functions and Associated Pathways	Cancer Implications	Therapy Approaches	References
FACT Complex	NRF2/KEAP1 pathway, ATR/CHK1 pathway	Breast Cancer, Hepatocellular Carcinoma, etc.	Curaxin	[20,21,22,23,24,25,26,27,28,29,30,31,32,33,34]
ASF1	GM-CSF dependent inflammatory pathway, Wnt signaling pathway	Lung Adenocarcinoma, Gastrointestinal Cancer, etc.	Chimeric Inhibitor	[35,36,37,38,39,40,41,42,43,44,45,46,47,48,49,50,51,52,53]
APLF	NHEJ pathway	Glioblastoma Multiforme, Bladder Cancer, etc.	N/A	[54,55,56,57,58,59,60,61,62,63,64,65,66,67]
NPM1	DDT pathway	Oral Squamous Cell Carcinoma, Acute Myeloid Leukemia, etc.	NSC348884 and RNAi	[68,69,70,71,72,73,74,75,76,77,78,79,80,81,82,83,84,85,86]
CHAF1A	Folate metabolism pathway	Diffuse Large B-Cell Carcinoma, Gastric Cancer, etc.	Knockout	[87,88,89,90,91,92]
CHAF1B	???	Squamous Cell Carcinoma, Larynx Carcinoma, etc.		[93,94,95,96,97,98]
P48	NF-κB pathway, HDAC pathway	Cervical Cancer, Anaplastic Thyroid Carcinoma, etc.		[99,100,101,102,103,104,105,106]
HIRA	MYC Pathway, Cell cycle pathway	Hereditary Leiomyomatosis and Renal Cell Carcinoma, Chronic Myeloid Leukemia, etc.	N/A	[107,108,109,110,111,112,113,114,115,116,117,118]
NAP1	JNK signaling pathway	Glioma, Ovarian Cancer, etc.	Gene Therapy	[119,120,121,122,123,124,125,126,127,128]
SPT6	hTERT signaling pathway	Colorectal Cancer, etc.	Chaetocin	[129,130,131,132,133,134]
DAXX	Epigenetic silencing pathway, Autophagy pathway	Pancreatic Neuroendocrine Tumors, Prostate Cancer, etc.	N/A	[135,136,137,138,139,140,141,142,143,144]
C1QBP	???	breast cancer, renal cancer, etc.	???	[145,146,147,148,149]

Abbreviations: FACT Complex, facilitates chromatin transcription complex; ASF1, anti-silencing function 1; APLF, aprataxin and polynucleotide kinase-like factor; NPM1, nucleophosmin; CHAF1A, chromatin assembly factor 1 subunit A; CHAF1B, chromatin assembly factor 1 subunit B; HIRA, histone cell cycle regulator; NAP1, nucleosome assembly protein 1; SPT6, transcription elongation factor; DAXX, death domain-associated protein; C1QBP, complement 1 Q subcomponent-binding protein; ???, unknown.

## References

[1] Luger K., Mader A.W., Richmond R.K., Sargent D.F., Richmond T.J. (1997). Crystal structure of the nucleosome core particle at 2.8 A resolution. Nature.

[2] Davey C.A., Sargent D.F., Luger K., Maeder A.W., Richmond T.J. (2002). Solvent mediated interactions in the structure of the nucleosome core particle at 1.9 a resolution. J. Mol. Biol..

[3] Bottomley M.J. (2004). Structures of protein domains that create or recognize histone modifications. EMBO Rep..

[4] Bowman G.D., Poirier M.G. (2015). Post-Translational Modifications of Histones That Influence Nucleosome Dynamics. Chem. Rev..

[5] Allfrey V.G., Faulkner R., Mirsky A.E. (1964). Acetylation and Methylation of Histones and Their Possible Role in the Regulation of Rna Synthesis. Proc. Natl. Acad. Sci. USA.

[6] Laskey R.A., Honda B.M., Mills A.D., Finch J.T. (1978). Nucleosomes are assembled by an acidic protein which binds histones and transfers them to DNA. Nature.

[7] De Koning L., Corpet A., Haber J.E., Almouzni G. (2007). Histone chaperones: An escort network regulating histone traffic. Nat. Struct. Mol. Biol..

[8] Hammond C.M., Stromme C.B., Huang H., Patel D.J., Groth A. (2017). Histone chaperone networks shaping chromatin function. Nat. Rev. Mol. Cell Biol..

[9] Loyola A., Almouzni G. (2007). Marking histone H3 variants: How, when and why?. Trends Biochem. Sci..

[10] Scott W.A., Campos E.I. (2020). Interactions With Histone H3 & Tools to Study Them. Front. Cell Dev. Biol..

[11] Bobde R.C., Saharan K., Baral S., Gandhi S., Samal A., Sundaram R., Kumar A., Singh A.K., Datta A., Vasudevan D. (2021). In Vitro Characterization of Histone Chaperones using Analytical, Pull-Down and Chaperoning Assays. J. Vis. Exp..

[12] Eitoku M., Sato L., Senda T., Horikoshi M. (2008). Histone chaperones: 30 years from isolation to elucidation of the mechanisms of nucleosome assembly and disassembly. Cell. Mol. Life Sci..

[13] Zhou C.Y., Johnson S.L., Gamarra N.I., Narlikar G.J. (2016). Mechanisms of ATP-Dependent Chromatin Remodeling Motors. Annu. Rev. Biophys..

[14] Das C., Lucia M.S., Hansen K.C., Tyler J.K. (2009). CBP/p300-mediated acetylation of histone H3 on lysine 56. Nature.

[15] Das C., Tyler J.K. (2013). Histone exchange and histone modifications during transcription and aging. Biochim. Biophys. Acta.

[16] Pchelintsev N.A., McBryan T., Rai T.S., van Tuyn J., Ray-Gallet D., Almouzni G., Adams P.D. (2013). Placing the HIRA histone chaperone complex in the chromatin landscape. Cell Rep..

[17] Karatas H., Townsend E.C., Cao F., Chen Y., Bernard D., Liu L., Lei M., Dou Y., Wang S. (2013). High-affinity, small-molecule peptidomimetic inhibitors of MLL1/WDR5 protein-protein interaction. J. Am. Chem. Soc..

[18] Seol J.H., Song T.Y., Oh S.E., Jo C., Choi A., Kim B., Park J., Hong S., Song I., Jung K.Y. (2015). Identification of small molecules that inhibit the histone chaperone Asf1 and its chromatin function. BMB Rep..

[19] Debela D.T., Muzazu S.G., Heraro K.D., Ndalama M.T., Mesele B.W., Haile D.C., Kitui S.K., Manyazewal T. (2021). New approaches and procedures for cancer treatment: Current perspectives. SAGE Open Med..

[20] Marciano G., Da Vela S., Tria G., Svergun D.I., Byron O., Huang D.T. (2018). Structure-specific recognition protein-1 (SSRP1) is an elongated homodimer that binds histones. J. Biol. Chem..

[21] Foglia B., Parola M. (2020). Of FACT complex and oxidative stress response: A KEAP1/NRF2-dependent novel mechanism sustaining hepatocellular carcinoma progression. Gut.

[22] Prendergast L., Hong E., Safina A., Poe D., Gurova K. (2020). Histone chaperone FACT is essential to overcome replication stress in mammalian cells. Oncogene.

[23] Bhakat K.K., Ray S. (2022). The FAcilitates Chromatin Transcription (FACT) complex: Its roles in DNA repair and implications for cancer therapy. DNA Repair..

[24] Chang H.W., Nizovtseva E.V., Razin S.V., Formosa T., Gurova K.V., Studitsky V.M. (2019). Histone Chaperone FACT and Curaxins: Effects on Genome Structure and Function. J. Cancer Metastasis Treat..

[25] Samant H., Amiri H.S., Zibari G.B. (2021). Addressing the worldwide hepatocellular carcinoma: Epidemiology, prevention and management. J. Gastrointest. Oncol..

[26] Chen Z., Han F., Du Y., Shi H., Zhou W. (2023). Hypoxic microenvironment in cancer: Molecular mechanisms and therapeutic interventions. Signal Transduct. Target. Ther..

[27] Rinaldi L., Vetrano E., Rinaldi B., Galiero R., Caturano A., Salvatore T., Sasso F.C. (2021). HCC and Molecular Targeting Therapies: Back to the Future. Biomedicines.

[28] Lukasiewicz S., Czeczelewski M., Forma A., Baj J., Sitarz R., Stanislawek A. (2021). Breast Cancer-Epidemiology, Risk Factors, Classification, Prognostic Markers, and Current Treatment Strategies—An Updated Review. Cancers.

[29] Khella C.A., Mehta G.A., Mehta R.N., Gatza M.L. (2021). Recent Advances in Integrative Multi-Omics Research in Breast and Ovarian Cancer. J. Pers. Med..

[30] Saldivar J.C., Cortez D., Cimprich K.A. (2017). The essential kinase ATR: Ensuring faithful duplication of a challenging genome. Nat. Rev. Mol. Cell Biol..

[31] Shen J., Yang C., Zhang M.S., Chin D.W., Chan F.F., Law C.T., Wang G., Cheng C.L., Chen M., Wan R.T. (2022). Histone chaperone FACT complex coordinates with HIF to mediate an expeditious transcription program to adapt to poorly oxygenated cancers. Cell Rep..

[32] Zhao Z., Cai Z., Jiang T., Han J., Zhang B. (2022). Histone Chaperones and Digestive Cancer: A Review of the Literature. Cancers.

[33] Volokh O.I., Sivkina A.L., Moiseenko A.V., Popinako A.V., Karlova M.G., Valieva M.E., Kotova E.Y., Kirpichnikov M.P., Formosa T., Studitsky V.M. (2022). Mechanism of curaxin-dependent nucleosome unfolding by FACT. Front. Mol. Biosci..

[34] Fleyshman D., Prendergast L., Safina A., Paszkiewicz G., Commane M., Morgan K., Attwood K., Gurova K. (2017). Level of FACT defines the transcriptional landscape and aggressive phenotype of breast cancer cells. Oncotarget.

[35] Wang X., Wang L., Dou J., Yu T., Cao P., Fan N., Borjigin U., Nashun B. (2021). Distinct role of histone chaperone Asf1a and Asf1b during fertilization and pre-implantation embryonic development in mice. Epigenetics Chromatin.

[36] Paul P.K., Rabaglia M.E., Wang C.Y., Stapleton D.S., Leng N., Kendziorski C., Lewis P.W., Keller M.P., Attie A.D. (2016). Histone chaperone ASF1B promotes human beta-cell proliferation via recruitment of histone H3.3. Cell Cycle.

[37] Messiaen S., Guiard J., Aigueperse C., Fliniaux I., Tourpin S., Barroca V., Allemand I., Fouchet P., Livera G., Vernet M. (2016). Loss of the histone chaperone ASF1B reduces female reproductive capacity in mice. Reproduction.

[38] Mbianda J., Bakail M., Andre C., Moal G., Perrin M.E., Pinna G., Guerois R., Becher F., Legrand P., Traore S. (2021). Optimal anchoring of a foldamer inhibitor of ASF1 histone chaperone through backbone plasticity. Sci. Adv..

[39] Zhu H., Ouyang H., Pan X., Zhang Z., Tan J., Yu N., Li M., Zhao Y. (2022). Increased ASF1B Expression Correlates With Poor Prognosis in Patients With Gliomas. Front. Oncol..

[40] Liang X., Yuan X., Yu J., Wu Y., Li K., Sun C., Li S., Shen L., Kong F., Jia J. (2017). Histone Chaperone ASF1A Predicts Poor Outcomes for Patients With Gastrointestinal Cancer and Drives Cancer Progression by Stimulating Transcription of beta-Catenin Target Genes. eBioMedicine.

[41] Myers D.J., Wallen J.M. (2024). Lung Adenocarcinoma. StatPearls.

[42] Li F., Huang Q., Luster T.A., Hu H., Zhang H., Ng W.L., Khodadadi-Jamayran A., Wang W., Chen T., Deng J. (2020). In Vivo Epigenetic CRISPR Screen Identifies Asf1a as an Immunotherapeutic Target in Kras-Mutant Lung Adenocarcinoma. Cancer Discov..

[43] Watterson A., Coelho M.A. (2023). Cancer immune evasion through KRAS and PD-L1 and potential therapeutic interventions. Cell Commun. Signal.

[44] Kumar A., Taghi Khani A., Sanchez Ortiz A., Swaminathan S. (2022). GM-CSF: A Double-Edged Sword in Cancer Immunotherapy. Front. Immunol..

[45] Brettingham-Moore K.H., Sprod O.R., Chen X., Oakford P., Shannon M.F., Holloway A.F. (2008). Determinants of a transcriptionally competent environment at the GM-CSF promoter. Nucleic Acids Res..

[46] Zhang W., Gao Z., Guan M., Liu N., Meng F., Wang G. (2021). ASF1B Promotes Oncogenesis in Lung Adenocarcinoma and Other Cancer Types. Front. Oncol..

[47] Bellelli R., Belan O., Pye V.E., Clement C., Maslen S.L., Skehel J.M., Cherepanov P., Almouzni G., Boulton S.J. (2018). POLE3-POLE4 Is a Histone H3-H4 Chaperone that Maintains Chromatin Integrity during DNA Replication. Mol. Cell.

[48] Shi L., Wang S., Zangari M., Xu H., Cao T.M., Xu C., Wu Y., Xiao F., Liu Y., Yang Y. (2010). Over-expression of CKS1B activates both MEK/ERK and JAK/STAT3 signaling pathways and promotes myeloma cell drug-resistance. Oncotarget.

[49] Fei-Zhang D.J., Moazzam Z., Ejaz A., Cloyd J., Dillhoff M., Beane J., Bentrem D.J., Pawlik T.M. (2023). The impact of digital inequities on gastrointestinal cancer disparities in the United States. J. Surg. Oncol..

[50] Jardim S.R., de Souza L.M.P., de Souza H.S.P. (2023). The Rise of Gastrointestinal Cancers as a Global Phenomenon: Unhealthy Behavior or Progress?. Int. J. Environ. Res. Public Health.

[51] Lecarpentier Y., Schussler O., Hebert J.L., Vallee A. (2019). Multiple Targets of the Canonical WNT/beta-Catenin Signaling in Cancers. Front. Oncol..

[52] Chen C., Bao H., Lin W., Chen X., Huang Y., Wang H., Yang Y., Liu J., Lv X., Teng L. (2022). ASF1b is a novel prognostic predictor associated with cell cycle signaling pathway in gastric cancer. J. Cancer.

[53] Miknis G.F., Stevens S.J., Smith L.E., Ostrov D.A., Churchill M.E. (2015). Development of novel Asf1-H3/H4 inhibitors. Bioorg. Med. Chem. Lett..

[54] Cherry A.L., Nott T.J., Kelly G., Rulten S.L., Caldecott K.W., Smerdon S.J. (2015). Versatility in phospho-dependent molecular recognition of the XRCC1 and XRCC4 DNA-damage scaffolds by aprataxin-family FHA domains. DNA Repair..

[55] Mehrotra P.V., Ahel D., Ryan D.P., Weston R., Wiechens N., Kraehenbuehl R., Owen-Hughes T., Ahel I. (2011). DNA repair factor APLF is a histone chaperone. Mol. Cell.

[56] Corbeski I., Dolinar K., Wienk H., Boelens R., van Ingen H. (2018). DNA repair factor APLF acts as a H2A-H2B histone chaperone through binding its DNA interaction surface. Nucleic Acids Res..

[57] Corbeski I., Guo X., Eckhardt B.V., Fasci D., Wiegant W., Graewert M.A., Vreeken K., Wienk H., Svergun D.I., Heck A.J.R. (2022). Chaperoning of the histone octamer by the acidic domain of DNA repair factor APLF. Sci. Adv..

[58] Hanif F., Muzaffar K., Perveen K., Malhi S.M., Simjee Sh U. (2017). Glioblastoma Multiforme: A Review of its Epidemiology and Pathogenesis through Clinical Presentation and Treatment. Asian Pac. J. Cancer Prev..

[59] Yalamarty S.S.K., Filipczak N., Li X., Subhan M.A., Parveen F., Ataide J.A., Rajmalani B.A., Torchilin V.P. (2023). Mechanisms of Resistance and Current Treatment Options for Glioblastoma Multiforme (GBM). Cancers.

[60] Fernandes C., Costa A., Osorio L., Lago R.C., Linhares P., Carvalho B., Caeiro C., De Vleeschouwer S. (2017). Current Standards of Care in Glioblastoma Therapy. Glioblastoma.

[61] Dong W., Li L., Teng X., Yang X., Si S., Chai J. (2020). End Processing Factor APLF Promotes NHEJ Efficiency and Contributes to TMZ- and Ionizing Radiation-Resistance in Glioblastoma Cells. Onco Targets Ther..

[62] Grundy G.J., Rulten S.L., Zeng Z., Arribas-Bosacoma R., Iles N., Manley K., Oliver A., Caldecott K.W. (2013). APLF promotes the assembly and activity of non-homologous end joining protein complexes. EMBO J..

[63] Khochikar M.V. (2011). Rationale for an early detection program for bladder cancer. Indian. J. Urol..

[64] Gupta R., Khan S.M., Mahajan M., Sharma P., Mahajan A. (2023). Urinary Bladder Carcinoma in Females: A Clinico-Pathological Assessment. Cureus.

[65] Viveiros N., Flores B.C., Lobo J., Martins-Lima C., Cantante M., Lopes P., Deantonio C., Palu C., Sainson R.C., Henrique R. (2022). Detailed bladder cancer immunoprofiling reveals new clues for immunotherapeutic strategies. Clin. Transl. Immunol..

[66] Minoli M., Kiener M., Thalmann G.N., Kruithof-de Julio M., Seiler R. (2020). Evolution of Urothelial Bladder Cancer in the Context of Molecular Classifications. Int. J. Mol. Sci..

[67] Richter C., Marquardt S., Li F., Spitschak A., Murr N., Edelhauser B.A.H., Iliakis G., Putzer B.M., Logotheti S. (2019). Rewiring E2F1 with classical NHEJ via APLF suppression promotes bladder cancer invasiveness. J. Exp. Clin. Cancer Res..

[68] Porwit A., McCullough J., Erber W.N. (2011). Blood and Bone Marrow Pathology.

[69] Senapati P., Bhattacharya A., Das S., Dey S., Sudarshan D., Vishwakarma J., Sudevan S., Ramachandran R., Maliekal T.T., Kundu T.K. (2022). Histone Chaperone Nucleophosmin Regulates Transcription of Key Genes Involved in Oral Tumorigenesis. Mol. Cell. Biol..

[70] Lindstrom M.S. (2011). NPM1/B23: A Multifunctional Chaperone in Ribosome Biogenesis and Chromatin Remodeling. Biochem. Res. Int..

[71] Chin L., Wong C.Y.G., Gill H. (2023). Targeting and Monitoring Acute Myeloid Leukaemia with Nucleophosmin-1 (NPM1) Mutation. Int. J. Mol. Sci..

[72] Laliberte C., Ng N., Eymael D., Higgins K., Ali A., Kiss A., Bradley G., Magalhaes M.A.O. (2021). Characterization of Oral Squamous Cell Carcinoma Associated Inflammation: A Pilot Study. Front. Oral. Health.

[73] Siegel R.L., Miller K.D., Jemal A. (2018). Cancer statistics, 2018. CA Cancer J. Clin..

[74] Warnakulasuriya S. (2009). Global epidemiology of oral and oropharyngeal cancer. Oral. Oncol..

[75] Vakiti A., Mewawalla P. Acute Myeloid Leukemia. https://www.statpearls.com/point-of-care/25443.

[76] Leukemia—Acute Myeloid—AML: Statistics. https://www.cancer.net/cancer-types/leukemia-acute-myeloid-aml/statistics.

[77] Medeiros B.C., Chan S.M., Daver N.G., Jonas B.A., Pollyea D.A. (2019). Optimizing survival outcomes with post-remission therapy in acute myeloid leukemia. Am. J. Hematol..

[78] Pollyea D.A., Altman J.K., Assi R., Bixby D., Fathi A.T., Foran J.M., Gojo I., Hall A.C., Jonas B.A., Kishtagari A. (2023). Acute Myeloid Leukemia, Version 3.2023, NCCN Clinical Practice Guidelines in Oncology. J. Natl. Compr. Cancer Netw..

[79] Falini B., Nicoletti I., Martelli M.F., Mecucci C. (2007). Acute myeloid leukemia carrying cytoplasmic/mutated nucleophosmin (NPMc+ AML): Biologic and clinical features. Blood.

[80] Falini B., Mecucci C., Tiacci E., Alcalay M., Rosati R., Pasqualucci L., La Starza R., Diverio D., Colombo E., Santucci A. (2005). Cytoplasmic nucleophosmin in acute myelogenous leukemia with a normal karyotype. N. Engl. J. Med..

[81] Federici L., Falini B. (2013). Nucleophosmin mutations in acute myeloid leukemia: A tale of protein unfolding and mislocalization. Protein Sci..

[82] Sekhar K.R., Freeman M.L. (2023). Nucleophosmin Plays a Role in Repairing DNA Damage and Is a Target for Cancer Treatment. Cancer Res..

[83] Ziv O., Zeisel A., Mirlas-Neisberg N., Swain U., Nevo R., Ben-Chetrit N., Martelli M.P., Rossi R., Schiesser S., Canman C.E. (2014). Identification of novel DNA-damage tolerance genes reveals regulation of translesion DNA synthesis by nucleophosmin. Nat. Commun..

[84] Rechkoblit O., Johnson R.E., Buku A., Prakash L., Prakash S., Aggarwal A.K. (2019). Structural insights into mutagenicity of anticancer nucleoside analog cytarabine during replication by DNA polymerase eta. Sci. Rep..

[85] Balusu R., Fiskus W., Rao R., Chong D.G., Nalluri S., Mudunuru U., Ma H., Chen L., Venkannagari S., Ha K. (2011). Targeting levels or oligomerization of nucleophosmin 1 induces differentiation and loss of survival of human AML cells with mutant NPM1. Blood.

[86] Zhou Y., Fang Y., Zhou J., Liu Y., Wu S., Xu B. (2021). NPM1 is a Novel Therapeutic Target and Prognostic Biomarker for Ewing Sarcoma. Front. Genet..

[87] Rawla P., Barsouk A. (2019). Epidemiology of gastric cancer: Global trends, risk factors and prevention. Przegląd Gastroenterol..

[88] Zheng L., Liang X., Li S., Li T., Shang W., Ma L., Jia X., Shao W., Sun P., Chen C. (2018). CHAF1A interacts with TCF4 to promote gastric carcinogenesis via upregulation of c-MYC and CCND1 expression. eBioMedicine.

[89] Wang D., Li X., Shen B., Chen X., Shu Y. (2019). Histone chaperone CHAF1A impacts the outcome of fluoropyrimidines-based adjuvant therapy in gastric cancer by regulating the expression of thymidylate synthetase. Gene.

[90] Padala S.A. Diffuse Large B-Cell Lymphoma. https://www.statpearls.com/point-of-care/24581.

[91] Wang L., Li L.R., Young K.H. (2020). New agents and regimens for diffuse large B cell lymphoma. J. Hematol. Oncol..

[92] Yan W., Shi X., Wang H., Liao A., Yang W. (2022). Aberrant SPOP-CHAF1A ubiquitination axis triggers tumor autophagy that endows a therapeutical vulnerability in diffuse large B cell lymphoma. J. Transl. Med..

[93] Koroulakis A. Laryngeal Cancer. https://www.statpearls.com/point-of-care/24035.

[94] Zhang W., Zeng W., Jiang A., He Z., Shen X., Dong X., Feng J., Lu H. (2021). Global, regional and national incidence, mortality and disability-adjusted life-years of skin cancers and trend analysis from 1990 to 2019: An analysis of the Global Burden of Disease Study 2019. Cancer Med..

[95] Mascolo M., Ilardi G., Merolla F., Russo D., Vecchione M.L., De Rosa G., Staibano S. (2012). Tissue microarray-based evaluation of Chromatin Assembly Factor-1 (CAF-1)/p60 as tumour prognostic marker. Int. J. Mol. Sci..

[96] Dotto G.P., Rustgi A.K. (2016). Squamous Cell Cancers: A Unified Perspective on Biology and Genetics. Cancer Cell.

[97] Sun Q., Fang Q., Guo S. (2015). A comparison of oral squamous cell carcinoma between young and old patients in a single medical center in China. Int. J. Clin. Exp. Med..

[98] Staibano S., Mignogna C., Lo Muzio L., Mascolo M., Salvatore G., Di Benedetto M., Califano L., Rubini C., De Rosa G. (2007). Chromatin assembly factor-1 (CAF-1)-mediated regulation of cell proliferation and DNA repair: A link with the biological behaviour of squamous cell carcinoma of the tongue?. Histopathology.

[99] Fowler J.R., Maani E.V., Dunton C.J., Gasalberti D.P., Jack B.W. (2024). Cervical Cancer. StatPearls.

[100] Stelzle D., Tanaka L.F., Lee K.K., Ibrahim Khalil A., Baussano I., Shah A.S.V., McAllister D.A., Gottlieb S.L., Klug S.J., Winkler A.S. (2021). Estimates of the global burden of cervical cancer associated with HIV. Lancet Glob. Health.

[101] Kong L., Yu X.P., Bai X.H., Zhang W.F., Zhang Y., Zhao W.M., Jia J.H., Tang W., Zhou Y.B., Liu C.J. (2007). RbAp48 is a critical mediator controlling the transforming activity of human papillomavirus type 16 in cervical cancer. J. Biol. Chem..

[102] Nicolas E., Ait-Si-Ali S., Trouche D. (2001). The histone deacetylase HDAC3 targets RbAp48 to the retinoblastoma protein. Nucleic Acids Res..

[103] Johansson C., Jamal Fattah T., Yu H., Nygren J., Mossberg A.K., Schwartz S. (2015). Acetylation of intragenic histones on HPV16 correlates with enhanced HPV16 gene expression. Virology.

[104] Lee K., Anastasopoulou C., Chandran C., Cassaro S. (2024). Thyroid Cancer. StatPearls.

[105] Lee H., Kim S.Y., Kim S.M., Chang H.J., Lee Y.S., Park C.S., Chang H.S. (2020). Long-term survival of patients with anaplastic thyroid cancer after multimodal treatment. Transl. Cancer Res..

[106] Pozdeyev N., Berlinberg A., Zhou Q., Wuensch K., Shibata H., Wood W.M., Haugen B.R. (2015). Targeting the NF-kappaB Pathway as a Combination Therapy for Advanced Thyroid Cancer. PLoS ONE.

[107] Smith M., Smith M. (2023). Chapter 14—The regulatory genome and complex common diseases. The Regulatory Genome in Adaptation, Evolution, Development, and Disease.

[108] Lamour V., Lecluse Y., Desmaze C., Spector M., Bodescot M., Aurias A., Osley M.A., Lipinski M. (1995). A human homolog of the S. cerevisiae HIR1 and HIR2 transcriptional repressors cloned from the DiGeorge syndrome critical region. Hum. Mol. Genet..

[109] Goldberg A.D., Banaszynski L.A., Noh K.M., Lewis P.W., Elsaesser S.J., Stadler S., Dewell S., Law M., Guo X., Li X. (2010). Distinct factors control histone variant H3.3 localization at specific genomic regions. Cell.

[110] Rai T.S., Cole J.J., Nelson D.M., Dikovskaya D., Faller W.J., Vizioli M.G., Hewitt R.N., Anannya O., McBryan T., Manoharan I. (2014). HIRA orchestrates a dynamic chromatin landscape in senescence and is required for suppression of neoplasia. Genes Dev..

[111] Schmidt L.S., Linehan W.M. (2014). Hereditary leiomyomatosis and renal cell carcinoma. Int. J. Nephrol. Renov. Dis..

[112] Menko F.H., Maher E.R., Schmidt L.S., Middelton L.A., Aittomaki K., Tomlinson I., Richard S., Linehan W.M. (2014). Hereditary leiomyomatosis and renal cell cancer (HLRCC): Renal cancer risk, surveillance and treatment. Fam. Cancer.

[113] Valcarcel-Jimenez L., Rogerson C., Yong C., Schmidt C., Yang M., Cremades-Rodelgo M., Harle V., Offord V., Wong K., Mora A. (2022). HIRA loss transforms FH-deficient cells. Sci. Adv..

[114] Stine Z.E., Walton Z.E., Altman B.J., Hsieh A.L., Dang C.V. (2015). MYC, Metabolism, and Cancer. Cancer Discov..

[115] PDQ Adult Treatment Editorial Board (2002). Chronic Myelogenous Leukemia Treatment (PDQ(R)): Health Professional Version. PDQ Cancer Information Summaries.

[116] Jabbour E., Kantarjian H. (2020). Chronic myeloid leukemia: 2020 update on diagnosis, therapy and monitoring. Am. J. Hematol..

[117] Uhm J. (2023). Treatment after failure of frontline therapy of chronic myeloid leukemia in chronic phase including allogeneic hematopoietic stem cell transplantation. Blood Res..

[118] Majumder A., Dharan A.T., Baral I., Varghese P.C., Mukherjee A., Subhadradevi L., Narayanan G., Dutta D. (2019). Histone chaperone HIRA dictate proliferation vs differentiation of chronic myeloid leukemia cells. FASEB Bioadv..

[119] Al-Bassam J., Corbett K.D. (2012). alpha-Tubulin acetylation from the inside out. Proc. Natl. Acad. Sci. USA.

[120] Andrews A.J., Chen X., Zevin A., Stargell L.A., Luger K. (2010). The histone chaperone Nap1 promotes nucleosome assembly by eliminating nonnucleosomal histone DNA interactions. Mol. Cell.

[121] Chen Z., Xie Y., Luo H., Song Y., Que T., Hu R., Huang H., Luo K., Li C., Qin C. (2021). NAP1L1 promotes proliferation and chemoresistance in glioma by inducing CCND1/CDK4/CDK6 expression through its interaction with HDGF and activation of c-Jun. Aging.

[122] Friedmann D.R., Aguilar A., Fan J., Nachury M.V., Marmorstein R. (2012). Structure of the alpha-tubulin acetyltransferase, alphaTAT1, and implications for tubulin-specific acetylation. Proc. Natl. Acad. Sci. USA.

[123] Taal W., Bromberg J.E., van den Bent M.J. (2015). Chemotherapy in glioma. CNS Oncol..

[124] Arora T., Mullangi S. Epithelial Ovarian Cancer. https://www.statpearls.com/point-of-care/95586.

[125] Zhu J.W., Charkhchi P., Akbari M.R. (2022). Potential clinical utility of liquid biopsies in ovarian cancer. Mol. Cancer.

[126] Rampes S., Choy S.P. (2022). Early diagnosis of symptomatic ovarian cancer in primary care in the UK: Opportunities and challenges. Prim. Health Care Res. Dev..

[127] Yeung T.L., Leung C.S., Yip K.P., Au Yeung C.L., Wong S.T., Mok S.C. (2015). Cellular and molecular processes in ovarian cancer metastasis. A Review in the Theme: Cell and Molecular Processes in Cancer Metastasis. Am. J. Physiol. Cell Physiol..

[128] Xiaohua Z., Xie Y., Huang W., Chen Z., Guo S. (2022). NAP1L1 promotes tumor proliferation through HDGF/C-JUN signaling in ovarian cancer. BMC Cancer.

[129] Dronamraju R., Hepperla A.J., Shibata Y., Adams A.T., Magnuson T., Davis I.J., Strahl B.D. (2018). Spt6 Association with RNA Polymerase II Directs mRNA Turnover During Transcription. Mol. Cell.

[130] McCullough L., Connell Z., Petersen C., Formosa T. (2015). The Abundant Histone Chaperones Spt6 and FACT Collaborate to Assemble, Inspect, and Maintain Chromatin Structure in Saccharomyces cerevisiae. Genetics.

[131] Siegel R.L., Wagle N.S., Cercek A., Smith R.A., Jemal A. (2023). Colorectal cancer statistics, 2023. CA Cancer J. Clin..

[132] Hossain M.S., Karuniawati H., Jairoun A.A., Urbi Z., Ooi J., John A., Lim Y.C., Kibria K.M.K., Mohiuddin A.K.M., Ming L.C. (2022). Colorectal Cancer: A Review of Carcinogenesis, Global Epidemiology, Current Challenges, Risk Factors, Preventive and Treatment Strategies. Cancers.

[133] Zhou H., Zhu L., Song J., Wang G., Li P., Li W., Luo P., Sun X., Wu J., Liu Y. (2022). Liquid biopsy at the frontier of detection, prognosis and progression monitoring in colorectal cancer. Mol. Cancer.

[134] Diao C., Guo P., Yang W., Sun Y., Liao Y., Yan Y., Zhao A., Cai X., Hao J., Hu S. (2021). SPT6 recruits SND1 to co-activate human telomerase reverse transcriptase to promote colon cancer progression. Mol. Oncol..

[135] Drane P., Ouararhni K., Depaux A., Shuaib M., Hamiche A. (2010). The death-associated protein DAXX is a novel histone chaperone involved in the replication-independent deposition of H3.3. Genes Dev..

[136] Rapkin L.M., Ahmed K., Dulev S., Li R., Kimura H., Ishov A.M., Bazett-Jones D.P. (2015). The histone chaperone DAXX maintains the structural organization of heterochromatin domains. Epigenetics Chromatin.

[137] Carraro M., Hendriks I.A., Hammond C.M., Solis-Mezarino V., Volker-Albert M., Elsborg J.D., Weisser M.B., Spanos C., Montoya G., Rappsilber J. (2023). DAXX adds a de novo H3.3K9me3 deposition pathway to the histone chaperone network. Mol. Cell.

[138] Radu E.C., Saizu A.I., Grigorescu R.R., Croitoru A.E., Gheorghe C. (2018). Metastatic neuroendocrine pancreatic tumor—Case report. J. Med. Life.

[139] Ro C., Chai W., Yu V.E., Yu R. (2013). Pancreatic neuroendocrine tumors: Biology, diagnosis, and treatment. Chin. J. Cancer.

[140] Ueda H., Akiyama Y., Shimada S., Mogushi K., Serizawa M., Matsumura S., Mitsunori Y., Aihara A., Ban D., Ochiai T. (2018). Tumor suppressor functions of DAXX through histone H3.3/H3K9me3 pathway in pancreatic NETs. Endocr.-Relat. Cancer.

[141] Rawla P. (2019). Epidemiology of Prostate Cancer. World J. Oncol..

[142] Crawford E.D., Heidenreich A., Lawrentschuk N., Tombal B., Pompeo A.C.L., Mendoza-Valdes A., Miller K., Debruyne F.M.J., Klotz L. (2019). Androgen-targeted therapy in men with prostate cancer: Evolving practice and future considerations. Prostate Cancer Prostatic Dis..

[143] Buskin A., Scott E., Nelson R., Gaughan L., Robson C.N., Heer R., Hepburn A.C. (2023). Engineering prostate cancer in vitro: What does it take?. Oncogene.

[144] Puto L.A., Benner C., Hunter T. (2015). The DAXX co-repressor is directly recruited to active regulatory elements genome-wide to regulate autophagy programs in a model of human prostate cancer. Oncoscience.

[145] Wang J., Huang C.L., Zhang Y. (2022). Complement C1q Binding Protein (C1QBP): Physiological Functions, Mutation-Associated Mitochondrial Cardiomyopathy and Current Disease Models. Front. Cardiovasc. Med..

[146] Barna J., Dimen D., Puska G., Kovacs D., Csikos V., Olah S., Udvari E.B., Pal G., Dobolyi A. (2019). Complement component 1q subcomponent binding protein in the brain of the rat. Sci. Rep..

[147] Lin J., Bao X., Li X.D. (2021). A tri-functional amino acid enables mapping of binding sites for posttranslational-modification-mediated protein-protein interactions. Mol. Cell.

[148] Scully O.J., Shyamasundar S., Matsumoto K., Dheen S.T., Yip G.W., Bay B.H. (2023). C1QBP Mediates Breast Cancer Cell Proliferation and Growth via Multiple Potential Signalling Pathways. Int. J. Mol. Sci..

[149] Wang Y., Fu D., Su J., Chen Y., Qi C., Sun Y., Niu Y., Zhang N., Yue D. (2017). C1QBP suppresses cell adhesion and metastasis of renal carcinoma cells. Sci. Rep..

[150] Cheng L., Zhang X., Wang Y., Gan H., Xu X., Lv X., Hua X., Que J., Ordog T., Zhang Z. (2019). Chromatin Assembly Factor 1 (CAF-1) facilitates the establishment of facultative heterochromatin during pluripotency exit. Nucleic Acids Res..

[151] Rouillon C., Eckhardt B.V., Kollenstart L., Gruss F., Verkennis A.E.E., Rondeel I., Krijger P.H.L., Ricci G., Biran A., van Laar T. (2023). CAF-1 deposits newly synthesized histones during DNA replication using distinct mechanisms on the leading and lagging strands. Nucleic Acids Res..

[152] Ahmad A., Karim H. (2010). Mutation on WD Dipeptide Motifs of the p48 Subunit of Chromatin Assembly Factor-1 Causing Viability and Growth of DT40 Chicken B Cell Line. Indones. J. Chem..

[153] Kadyrova L.Y., Rodriges Blanko E., Kadyrov F.A. (2013). Human CAF-1-dependent nucleosome assembly in a defined system. Cell Cycle.

[154] Geis F.K., Sabo Y., Chen X., Li Y., Lu C., Goff S.P. (2022). CHAF1A/B mediate silencing of unintegrated HIV-1 DNAs early in infection. Proc. Natl. Acad. Sci. USA.

[155] Sykaras A.G., Pergaris A., Theocharis S. (2021). Challenging, Accurate and Feasible: CAF-1 as a Tumour Proliferation Marker of Diagnostic and Prognostic Value. Cancers.

[156] Pacifico F., Paolillo M., Chiappetta G., Crescenzi E., Arena S., Scaloni A., Monaco M., Vascotto C., Tell G., Formisano S. (2007). RbAp48 is a target of nuclear factor-kappaB activity in thyroid cancer. J. Clin. Endocrinol. Metab..

[157] Wang R., Zheng X., Zhang L., Zhou B., Hu H., Li Z., Zhang L., Lin Y., Wang X. (2017). Histone H4 expression is cooperatively maintained by IKKbeta and Akt1 which attenuates cisplatin-induced apoptosis through the DNA-PK/RIP1/IAPs signaling cascade. Sci. Rep..

[158] Verreault A., Kaufman P.D., Kobayashi R., Stillman B. (1996). Nucleosome assembly by a complex of CAF-1 and acetylated histones H3/H4. Cell.

[159] Chan F.F., Wong C.M. (2022). PP039 Inhibition of CAF-1 histone chaperone complex triggers cytosolic DNA and dsRNA sensing pathways and induces intrinsic immunity of hepatocellular carcinoma. ESMO Open.

[160] Gomes A.P., Ilter D., Low V., Rosenzweig A., Shen Z.J., Schild T., Rivas M.A., Er E.E., McNally D.R., Mutvei A.P. (2019). Dynamic Incorporation of Histone H3 Variants into Chromatin Is Essential for Acquisition of Aggressive Traits and Metastatic Colonization. Cancer Cell.

[161] Liu Y., Li X., Zhang Y., Tang Y., Fang W., Liu X., Liu Z. (2021). NAP1L1 targeting suppresses the proliferation of nasopharyngeal carcinoma. Biomed. Pharmacother..

[162] Obara E.A.A., Aguilar-Morante D., Rasmussen R.D., Frias A., Vitting-Serup K., Lim Y.C., Elbaek K.J., Pedersen H., Vardouli L., Jensen K.E. (2020). SPT6-driven error-free DNA repair safeguards genomic stability of glioblastoma cancer stem-like cells. Nat. Commun..

